# Combined Ozone Therapy and Negative Pressure Wound Therapy with Instillation for Charcot Neuroarthropathy-Associated Foot Wounds

**DOI:** 10.3390/jcm15218479

**Published:** 2026-10-30

**Authors:** Izabella Kuźmiuk-Glembin, Agnieszka Białomyzy, Daniel Kotrych, Michał Sadowski, Bogdan Biedunkiewicz, Leszek Tylicki, Tomasz Niewęgłowski

**Affiliations:** 1 Department of Nephrology, Transplantology and Internal Diseases, Medical University of Gdańsk, University Clinical Centre, Smoluchowskiego St. 17, 80-214 Gdansk, Poland; 2 ETER-MED, Wound Care Clinic, Non-Public Healthcare Centre, 80-822 Gdansk, Polandtn@etermed.pl (T.N.); 3 Department of Orthopaedic Oncology and Reconstructive Surgery, Hereditary Cancer Diagnosis and Treatment Center, Pomeranian Medical University of Szczecin, Unii Lubelskiej St 1, 71-252 Szczecin, Poland

**Keywords:** Charcot neuroarthropathy, diabetic foot, ozone therapy, negative-pressure wound therapy, wound healing, NPWTi-d, diabetic foot infection

## Abstract

**Background/Objectives**: Charcot neuro-osteoarthropathy (CNO) complicated by ulceration and infection represents a major limb-preservation challenge in diabetes-related foot disease. Evidence regarding the combined use of ozone therapy (OT) and negative pressure wound therapy with instillation and dwell time (NPWTi-d) remains limited. This study evaluated outcomes of a multimodal treatment protocol incorporating both modalities. **Methods**: This retrospective study included 30 patients with diabetes and CNO-associated foot wounds managed using a multidisciplinary protocol incorporating OT and NPWTi-d. The primary outcome was wound volume reduction, while secondary outcomes included pain, inflammatory biomarkers, microbiological findings, and safety. **Results**: Wound volume decreased significantly during follow-up, from a median of 8.0 cm^3^ at baseline to 1.5 cm^3^ after 3 weeks, with further reduction to 0 cm^3^ by week 6 (all *p* < 0.001). Significant improvements were observed in wound bed characteristics and exudate. Mean pain intensity (NRS) decreased from 6.9 ± 1.1 to 0.9 ± 1.1 points (*p* < 0.001). CRP and PCT concentrations declined significantly (both *p* < 0.001). Follow-up wound cultures demonstrated a lower frequency of isolation of several initially identified pathogens. No serious treatment-related adverse events occurred. **Conclusions**: This retrospective case series demonstrated favourable short-term changes in wound-related outcomes during a multimodal treatment strategy incorporating OT and NPWTi-d in patients with CNO-associated wounds. Given the uncontrolled study design and concurrent interventions, these findings should be considered exploratory and do not establish the independent efficacy of either modality or their combination.

## 1. Introduction

Despite substantial advances in multidisciplinary diabetic foot care, diabetes-related foot disease (DFD) remains a major source of morbidity, healthcare expenditure, and lower-extremity amputation worldwide. Approximately 18.6 million individuals are estimated to develop a diabetic foot ulcer (DFU) each year. The lifetime risk of DFU is estimated at 19–34%, while ulcer recurrence remains common, occurring in approximately 42% of patients within the first year after healing and in up to 65% within five years. Moreover, only 30–40% of DFUs achieve complete healing within 12 weeks despite contemporary treatment strategies, underscoring the substantial burden imposed by chronic wounds in individuals with diabetes [1,2,3].

Among the most severe manifestations of diabetes-related peripheral neuropathy is Charcot neuro-osteoarthropathy (CNO), a progressive and potentially limb-threatening disorder characterized by inflammatory destruction of bones, joints and surrounding soft tissues of the foot and ankle. Although traditionally regarded as an uncommon complication, increasing evidence suggests that CNO remains substantially underrecognized, particularly during its early stages. Its prevalence estimates vary considerably according to the population studied and the diagnostic methods applied. Recent population-based studies have reported prevalence rates of approximately 0.04–0.3% among individuals with diabetes, whereas substantially higher frequencies have been described in specialized diabetic foot services [4,5,6]. In a nationwide Danish cohort including more than 300,000 individuals with diabetes, the prevalence of CNO was estimated at 0.56%, with an incidence of 7.4 cases per 10,000 person-years, representing the largest population-based assessment currently available [7]. Bilateral involvement has been reported in approximately 9 up to 22% of affected individuals [8].

The pathophysiology of CNO extends beyond the traditional concept of neuropathic joint destruction and is currently understood as a complex interaction between peripheral neuropathy, repetitive mechanical stress and dysregulated inflammatory signalling. In the insensate foot, repetitive microtrauma may occur without protective sensory feedback, triggering an exaggerated local inflammatory response. Increased expression of pro-inflammatory cytokines, activation of the receptor activator of nuclear factor kappa-B, overexpression of RANKL pathway and enhanced genesis of osteoclasts collectively promote accelerated bone resorption and progressive skeletal instability [6,9,10]. These processes ultimately result in fragmentation, subluxation and collapse of the osseous architecture, leading to severe deformity, altered biomechanics and loss of functional integrity of the foot.

Contemporary literature increasingly recognizes CNO as a distinct high-risk manifestation of DFD rather than a rare orthopaedic complication. Recent perspectives have emphasized its strong association with ulceration, infection, functional impairment, and lower-extremity amputation, highlighting the need for earlier recognition and more proactive management strategies [4,11]. However, timely diagnosis remains challenging because active CNO often presents with non-specific clinical features, including swelling, erythema, and increased local temperature, which may mimic cellulitis, deep vein thrombosis, trauma, gout, or osteomyelitis [11]. As a result, diagnostic delay remains common and may allow ongoing skeletal destruction before effective off-loading is initiated. Current International Working Group on the Diabetic Foot (IWGDF) guidelines therefore strongly advocate prompt recognition and immediate immobilization to limit progressive structural collapse and preserve long-term foot function [12].

The clinical significance of CNO extends far beyond bone destruction. Progressive deformity alters plantar pressure distribution, increases focal mechanical stress and substantially predisposes patients to recurrent skin breakdown and ulceration. Previous studies have demonstrated that ulceration develops in a substantial proportion of patients with established Charcot deformities and represents a major determinant of adverse outcomes. Once ulceration occurs, the risk of deep infection and osteomyelitis increases considerably, creating a complex clinical scenario characterized by the coexistence of skeletal instability, chronic inflammation, bacterial contamination and impaired tissue repair. Several investigations have demonstrated that patients with concomitant CNO and ulceration experience markedly higher rates of hospitalization, lower-extremity amputation and healthcare utilization compared with diabetic patients without Charcot-related deformity [4,13]. Consequently, treatment goals increasingly extend beyond wound closure alone and focus on limb preservation, maintenance of ambulation and prevention of recurrent tissue loss.

In addition to conventional wound care, effective management of plantar diabetic foot ulcers requires correction or mitigation of the underlying biomechanical factors responsible for excessive plantar pressure. According to the IWGDF guidelines, non-removable knee-high offloading devices remain the preferred first-line intervention for neuropathic plantar forefoot and midfoot ulcers, whereas surgical offloading may be considered in selected patients with ulcers that fail to heal despite appropriate conservative treatment. Surgical options include Achilles tendon lengthening, metatarsal head resection, joint arthroplasty, and metatarsal osteotomy, depending on ulcer location and the underlying deformity. In particular, distal metatarsal osteotomies, including minimally invasive floating osteotomy techniques, have gained increasing attention for the management of persistent or recurrent neuropathic plantar ulcers beneath the lesser metatarsal heads [12,14]. These procedures aim to reduce focal plantar pressure by altering metatarsal head loading while minimizing soft-tissue disruption. Recent systematic reviews have reported high rates of ulcer healing and relatively low recurrence following minimally invasive metatarsal osteotomies [15,16]. Nevertheless, transfer lesions, infection, and non-union remain relevant complications, and the available evidence is predominantly derived from relatively small, non-randomized studies. Accordingly, careful patient selection and continued postoperative offloading remain essential.

The surgical management of CNO-associated ulceration requires additional consideration because the underlying pathology frequently involves complex osseous deformity, altered weight-bearing mechanics, and, in some cases, substantial joint instability. Unlike isolated neuropathic forefoot ulcers, wounds associated with established Charcot deformity may require exostectomy, correction of equinus deformity, reconstructive realignment, or stabilization of the affected foot segments to achieve durable pressure redistribution. The selection of surgical treatment depends on the anatomical distribution and activity of CNO, deformity severity, soft-tissue condition, vascular status, and the presence of infection. Consequently, minimally invasive distal metatarsal osteotomy should not be regarded as universally applicable to CNO-related plantar wounds, particularly those arising from midfoot collapse or rocker-bottom deformity. In this context, advanced local wound therapies may complement comprehensive multidisciplinary management by supporting wound-bed preparation and soft-tissue healing, but they do not replace adequate offloading, infection management, or corrective surgery when clinically indicated.

The treatment becomes substantially more challenging when CNO is complicated by chronic wounds, postoperative tissue defects, osteomyelitis or extensive soft tissue involvement. These patients frequently require prolonged multidisciplinary management involving orthopaedic surgeons, podiatrists, vascular specialists, infectious disease physicians and wound care teams. Despite advances in DFD care, outcomes remain suboptimal, highlighting the need for adjunctive therapeutic strategies capable of addressing the multiple mechanisms contributing to impaired healing [4,13].

Negative pressure wound therapy with instillation and dwell time (NPWTi-d) has become an established component of advanced wound management for complex DFU. Through the controlled application of subatmospheric pressure, NPWTi-d facilitates exudate removal, reduces tissue oedema, improves local perfusion and promotes granulation tissue formation. Meta-analyses have demonstrated favourable effects on wound healing outcomes and wound size reduction in selected diabetic foot populations [17,18].

Ozone therapy (OT) has emerged as another promising adjunctive modality in chronic wound care because of its broad-spectrum antimicrobial, immunomodulatory and oxygenation-enhancing properties. Ozone (O_3_) is a highly reactive triatomic form of oxygen with potential antimicrobial activity mediated by oxidative interactions with microbial membranes and other cellular components. Its biological effects are concentration- and exposure-dependent and may involve redox-sensitive pathways implicated in tissue responses to injury. Experimental and clinical evidence suggests that OT may contribute to bacterial clearance, modulation of inflammatory pathways and improvement of local tissue oxygen metabolism. Recent systematic reviews evaluating OT in DFU have reported encouraging effects on wound healing and infection control, although study heterogeneity remains substantial [19,20].

The rationale for combining OT with NPWTi-d lies in their potentially complementary mechanisms: NPWTi-d facilitates wound fluid removal, repeated instillation, and wound-bed preparation, while OT may provide additional local antimicrobial and redox-mediated effects. This approach was also informed by previously published clinical experience in an independent cohort of patients with diabetes-related foot wounds, although a specific additive or synergistic benefit of the combination remains unproven [21].

Despite increasing interest in both NPWTi-d and OT, evidence regarding their combined application in patients with CNO-associated wounds remains extremely limited. This knowledge gap is particularly relevant because CNO complicated by ulceration and infection represents one of the most challenging limb preservation scenarios encountered in DFD practice. Therefore, the present study aimed to perform an exploratory clinical evaluation of a multimodal treatment protocol combining OT and NPWTi-d in patients with CNO-associated foot wounds. The primary objective was to evaluate the clinical outcomes of this combined therapeutic approach in promoting wound healing. Secondary objectives included assessment of pain intensity, inflammatory biomarkers, clinical and microbiological indicators of infection and factors potentially associated with treatment response.

The novelty of the present study does not lie in the isolated application of OT or NPWTi-d as individual interventions, but rather in the real-world evaluation of their combined use within a structured limb-preservation protocol implemented in patients with CNO, a population characterized by severe deformity, chronic ulceration, recurrent infection and elevated risk of lower-extremity amputation. Given the retrospective and non-controlled design, the study was intended to generate preliminary clinical evidence regarding the feasibility, safety and potential therapeutic benefits of this combined treatment strategy.

## 2. Materials and Methods

### 2.1. Study Design and Patient Population

This retrospective observational cohort study was conducted at the Specialized Chronic Wound Care Centre between 2019 and 2022. The study evaluated the outcomes of a standardized multimodal treatment protocol combining OT and NPWTi-d with wound instillation in patients with diabetes mellitus and CNO-associated foot wounds. The study was approved by the Bioethics Committee of the Pomeranian Medical University (approval no. KB.006.130.2023; approval date: 20 December 2023) and was conducted in accordance with the ethical principles of the Declaration of Helsinki. Written informed consent for treatment and the use of anonymized clinical data for research purposes had been obtained from all participants.

The analysis included 30 consecutive patients treated during the study period. Eligible individuals had diabetes mellitus, CNO involving the foot, and chronic wounds requiring specialist wound care management. Most patients had previously undergone prolonged conventional treatment without satisfactory clinical improvement before referral to the study centre. Therefore, the analysed cohort reflects a real-world population of patients with advanced CNO-associated wounds managed in a tertiary wound care setting. Although CNO was diagnosed in routine clinical practice, standardized information regarding disease activity, anatomical involvement, radiological classification, duration of CNO, and previous Charcot-related surgery was not consistently available for retrospective analysis.

Vascular status was assessed during the initial clinical evaluation using the ankle–brachial index (ABI), transcutaneous oxygen pressure (TcPO_2_), and Doppler ultrasonography as part of routine clinical care. These investigations informed treatment eligibility and the assessment of limb perfusion. Patients with clinically significant limb-threatening ischemia requiring vascular intervention were not considered eligible for the local wound-treatment protocol. The hemodynamic measurements were interpreted in conjunction with the overall clinical and vascular assessment rather than as isolated diagnostic criteria for chronic limb-threatening ischemia (CLTI).

### 2.2. Treatment Protocol

All patients were managed according to a standardized multimodal wound care protocol routinely used at the study centre. The therapeutic strategy integrated surgical wound bed preparation, OT, NPWTi-d, systemic antimicrobial treatment when clinically indicated, and individualized off-loading interventions.

Treatment followed a stepwise, clinically individualized approach. As treatment components could overlap, the protocol should not be interpreted as a sequence of mutually exclusive interventions. Initial management consisted of comprehensive wound assessment and repeated sharp debridement aimed at removing necrotic tissue, infected soft tissue and non-viable bone structures whenever clinically indicated. Microbiological specimens were obtained before initiation of targeted antimicrobial therapy. Systemic antibiotics were prescribed according to clinical presentation, microbiological findings and current standards of care before OT and NPWTi-d was administered. Bone infection was assessed as part of routine clinical care, based on clinical presentation and available diagnostic findings. The diagnosis of osteomyelitis was established or suspected according to the overall clinical assessment, with diagnostic investigations individualized to each patient. Accordingly, the reported cases included both suspected and clinically documented bone infection.

Topical OT was administered using ozonated olive oil preparations applied directly to the wound surface during routine dressing changes. Ozonated olive oil was produced through repeated saturation of cold-pressed olive oil with ozone at a concentration of 70 μg/mL. NPWTi-d was performed using a closed-system device equipped with an instillation and drainage function (GENADYNA^®^, Hicksville, NY, USA) incorporating a multifunctional access port (Banasiewicz port). Ozonated saline solution was prepared immediately before treatment by saturating 500 mL of 0.9% sodium chloride solution with an oxygen–ozone mixture at a target ozone concentration of 70 μg/mL for 30 min using a medical ozone generator (ATO-3).

Each NPWTi-d treatment cycle consisted of three consecutive stages: instillation of ozonated saline into the wound bed until complete saturation of the polyurethane foam dressing, a dwell period of approximately 5–10 min allowing interaction between the solution and wound tissues, and subsequent evacuation of fluid under negative pressure. Particular attention was paid to maintaining treatment within the period of maximal antimicrobial activity of dissolved ozone.

Negative pressure levels were maintained between −100 and −125 mmHg throughout treatment. Instillation therapy was performed once daily for 10 consecutive days. Following the initial 10-day instillation phase, conventional NPWT was continued as clinically indicated, with treatment duration individualized according to wound characteristics and clinical response.

Between NPWTi-d sessions, wounds were protected using dressings impregnated with ozonated olive oil. The complete treatment protocol, including surgical debridement, ozone-based interventions and NPWTi-d, was implemented over a six-week observation period. Dressings were changed every three days in 23 patients (76.7%) and every four days in seven patients (23.3%).

Conventional NPWT was incorporated into the sequential wound-management protocol and did not constitute a separate comparator group.

Mechanical offloading and mobility support were individualized according to patients’ functional limitations and clinical needs. Mobility aids documented at baseline included crutches, walking frames and wheelchairs; these categories were not mutually exclusive. However, the specific therapeutic offloading devices used throughout treatment and adherence to prescribed offloading were not systematically recorded and could not be reliably assessed retrospectively.

### 2.3. Outcome Measures

Clinical outcomes were evaluated at baseline and after 3 and 6 weeks of treatment. The primary outcome was wound healing assessed through changes in wound volume during follow-up. Secondary outcomes included changes in pain intensity, inflammatory biomarkers, wound bed characteristics, wound exudate parameters, clinical signs of infection, microbiological findings and treatment safety with particular attention to serious events considered related to the treatment protocol.

Given the exploratory nature of the study, no predefined efficacy thresholds were established. All outcomes were analysed longitudinally to assess changes occurring during treatment.

### 2.4. Clinical and Laboratory Assessment

Clinical wound assessment was performed during routine follow-up visits using a standardized evaluation protocol. Wound volume was calculated using measurements of maximal wound length, width and depth and expressed in cubic centimetres (cm^3^). To minimize measurement variability, all assessments were performed using the same standardized methodology throughout the study period by the same investigator. Although the same measurement approach was applied at each assessment, this method may overestimate the actual volume of irregularly shaped wounds. The wound bed was evaluated according to the predominant stage of healing, including inflammatory/destructive, proliferative and remodelling phases. The presence of necrotic tissue, granulation tissue formation and epithelialization was documented during each assessment. Wound exudate was evaluated qualitatively with regard to both quantity and appearance. Clinical descriptors included purulent, dense, cloudy, serous, scant or absent exudate.

Pain intensity was assessed using the Numeric Rating Scale (NRS), ranging from 0 (no pain) to 10 (worst imaginable pain), based on clinical assessments documented at baseline and at weeks 3 and 6. The available records did not consistently distinguish wound-related, neuropathic, and musculoskeletal pain or specify whether NRS scores were obtained at rest or during wound-care procedures. Analgesic treatment was not modified as part of the study protocol; however, detailed medication regimens and potential changes outside the protocol were not systematically recorded.

Systemic inflammatory activity was assessed using serum concentrations of C-reactive protein (CRP) and procalcitonin (PCT). Glycaemic control was evaluated using glycated haemoglobin (HbA1c) values obtained from the clinical records.

Clinical signs of local and systemic infection, including erythema, oedema, purulent discharge, malodour and fistula formation, were recorded throughout treatment.

### 2.5. Microbiological Assessment

Microbiological assessment was performed as part of routine clinical wound care, with wound specimens obtained for pathogen identification and to guide antimicrobial management. Microbiological findings were assessed at baseline and at the six-week follow-up in all 30 patients.

Samples were processed according to standard laboratory procedures, and bacterial isolates were identified by the local microbiology laboratory. The retrospective nature of the study did not allow consistent verification of sampling techniques across all assessments. Antibiotic treatment was administered when clinically indicated and individualized according to microbiological findings and clinical judgment. Antibiotic selection and treatment duration were determined by the treating physicians as part of routine multidisciplinary wound management. Surgical debridement and local wound care were performed as clinically indicated. Microbiological outcomes were assessed descriptively based on the presence or absence of identified pathogens rather than quantitative measurements of bacterial load.

### 2.6. Statistical Analysis

Statistical analyses were performed using Stata/BE version 18 (StataCorp LLC, College Station, TX, USA). Continuous variables were summarized as mean ± standard deviation (SD) or median with interquartile range (IQR), depending on data distribution. Categorical variables were presented as absolute frequencies and percentages. The distribution of continuous variables was assessed using the Shapiro–Wilk test. Because of the relatively small sample size and non-normal distribution of several variables, non-parametric statistical methods were used where appropriate.

Changes in continuous outcomes between baseline, week 3 and week 6 were analysed using the Wilcoxon signed-rank test. Categorical outcomes assessed repeatedly in the same patients were summarized descriptively using absolute frequencies and percentages at each assessment point, without inferential testing of longitudinal differences. The longitudinal assessment of continuous outcomes was based on pairwise within-patient comparisons using the Wilcoxon signed-rank test. These analyses were intended to describe changes between predefined assessment points rather than to evaluate an overall longitudinal effect. Given the exploratory nature of the study, no formal adjustment for multiple comparisons was applied, and the resulting *p*-values should be interpreted as descriptive and hypothesis-generating.

Exploratory associations between baseline characteristics and changes in wound volume and pain intensity from baseline to week 6 were examined using ordinary least-squares linear regression. Candidate variables included selected demographic, clinical, and laboratory characteristics. The laboratory model for wound-volume reduction included baseline CRP, PCT, and HbA1c concentrations (*n* = 30). Additional models examined demographic and clinical characteristics, while changes in pain intensity were evaluated separately. Given the limited sample size, these analyses were considered hypothesis-generating and were not intended for the development or validation of predictive models. Regression findings are presented as β coefficients with corresponding 95% confidence intervals and *p*-values.

All statistical tests were two-sided, and statistical significance was defined as a *p*-value < 0.05.

## 3. Results

### 3.1. Baseline Characteristics

The study cohort consisted of 30 patients with diabetes mellitus and CNO-associated foot wounds. There were 22 men (73.3%) and 8 women (26.7%), with a mean age of 64.2 ± 16.6 years (range: 21–87 years). All participants presented with impaired mobility at baseline. Ambulation most commonly required the use of crutches (73.3%), whereas 23.3% of patients used a wheelchair and 6.7% relied on a walking frame.

Type 1 diabetes was present in 20 patients (66.7%), while 10 patients (33.3%) had type 2 diabetes. Diabetic neuropathy and polyneuropathy were documented in 86.7% of the cohort. The study population was characterized by a high burden of cardiovascular comorbidities, including hypertension (80.0%), chronic coronary syndrome (46.7%), chronic heart failure (26.7%) and lower-extremity oedema (76.7%). Detailed comorbidity data are presented in Table 1. The relatively high proportion of patients with type 1 diabetes should be interpreted in the context of referral bias inherent to a tertiary wound care centre. Most participants required insulin therapy (66.7%), and baseline glycaemic control remained suboptimal, with a median HbA1c of 7.2% (IQR 6.4–8.6).

At baseline, the median ankle–brachial index was 1.0 (IQR 0.99–1.10), while the median transcutaneous oxygen pressure was 35 mmHg (IQR 30–50), reflecting the vascular characteristics of patients included in the study.

Baseline wound assessment revealed a substantial burden of chronic CNO-associated plantar ulceration. All wounds were located on the plantar aspect of the foot, and approximately two-thirds had persisted for more than one year before referral. The median estimated wound volume at baseline was 8.0 cm^3^ (IQR 2.4–19.2), while sensory disturbances were documented in 93.3% of patients. Wound severity was further characterized through wound-bed appearance, exudate characteristics, and clinical signs of infection. However, standardized ulcer severity scores were not consistently available in the retrospective documentation. The prolonged duration of ulceration and extensive history of previous interventions highlight the complexity of this patient population. Details of wound characteristics and treatments received before referral are presented in Table 2 and Table 3, respectively.

### 3.2. Wound Healing Outcomes

A progressive reduction in estimated wound volume was observed throughout the study period. Median wound volume decreased from 8.0 cm^3^ (IQR 2.4–19.2) at baseline to 1.5 cm^3^ (IQR 0.5–4.8) at week 3 and 0 cm^3^ (IQR 0–0.05) at week 6. Statistically significant reductions were observed between baseline and week 3, weeks 3 and 6, and baseline and week 6 (all *p* < 0.001). These findings demonstrate a progressive reduction in wound volume during the six-week observation period. Importantly, a median estimated wound volume of 0 cm^3^ at week 6 reflects the measurement-based assessment of wound dimensions and does not necessarily indicate complete epithelialization or definitive wound closure. These outcomes were not systematically assessed as separate endpoints, and individual percentage reductions in wound volume were not included in the original analysis.

Evaluation of wound bed characteristics demonstrated a progressive shift toward more advanced healing stages. At baseline, 27 patients (90.0%) were classified as stage I (destructive), two (6.7%) as stage 0 (prodromal), and one (3.3%) as stage II (reparative). By week 3, 23 patients (76.7%) had progressed to stage II, while seven (23.3%) remained at stage I. At week 6, 23 patients (76.7%) were classified as stage III (consolidation), and the remaining seven (23.3%) as stage II.

Similarly, wound exudate characteristics improved over time. At baseline, wounds were predominantly characterized by abundant purulent, dense and cloudy exudate. After 3 weeks of treatment, most wounds exhibited smaller volumes of exudate and a transition toward clearer wound drainage. By week 6, wounds were characterized predominantly by absent or minimal serous exudate.

No serious adverse events attributed to the multimodal treatment protocol were documented during the six-week observation period. Safety findings were derived from retrospective clinical records and therefore reflect events documented during routine patient care rather than systematic prospective adverse-event surveillance.

### 3.3. Pain Assessment

**Figure 1:**
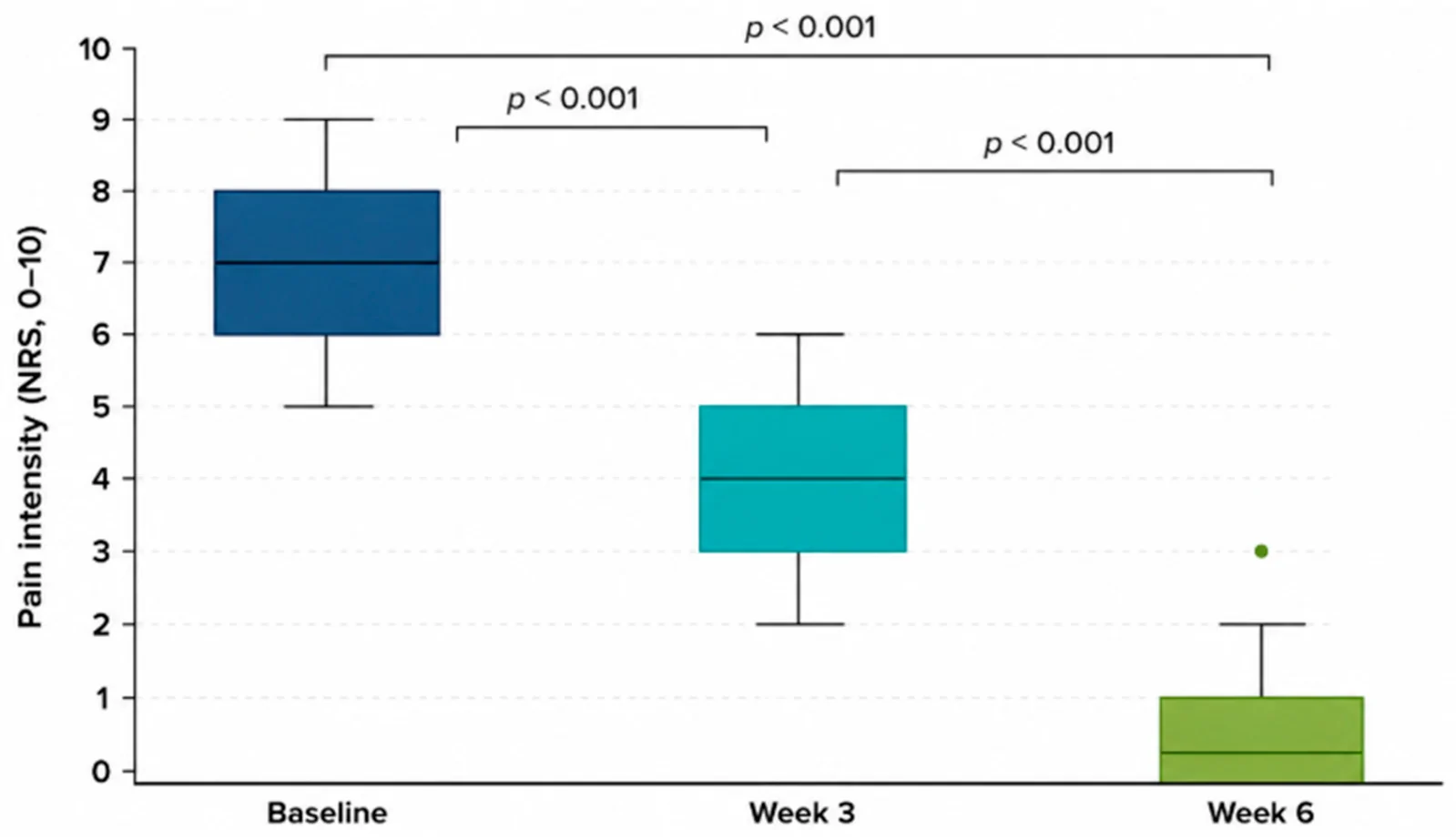
Changes in pain intensity measured using the Numeric Rating Scale (NRS) during the 6-week treatment period. NRS—Numeric Rating Scale (0–10); Statistical Analysis: Wilcoxon signed-rank test.

Pain intensity improved significantly throughout treatment. Mean NRS scores decreased from 6.9 ± 1.1 points at baseline to 3.9 ± 1.0 points after 3 weeks and to 0.9 ± 1.1 points at week 6. Statistically significant reductions in pain intensity were observed between baseline and week 3 (*p* < 0.001), between week 3 and week 6 (*p* < 0.001), and over the entire observation period (*p* < 0.001) (Figure 1).

Pain characteristics also evolved during treatment. At baseline, 27 patients (90%) reported pain described as sharp or of moderate intensity. By week 3, intermittent pain predominated, whereas pulsatile pain was reported only occasionally. At week 6, no patients reported persistent pain, with only a minority experiencing intermittent or occasional pulsatile discomfort (*n* = 3 patients, 10%).

### 3.4. Inflammatory Biomarkers and Infection Control

**Figure 2:**
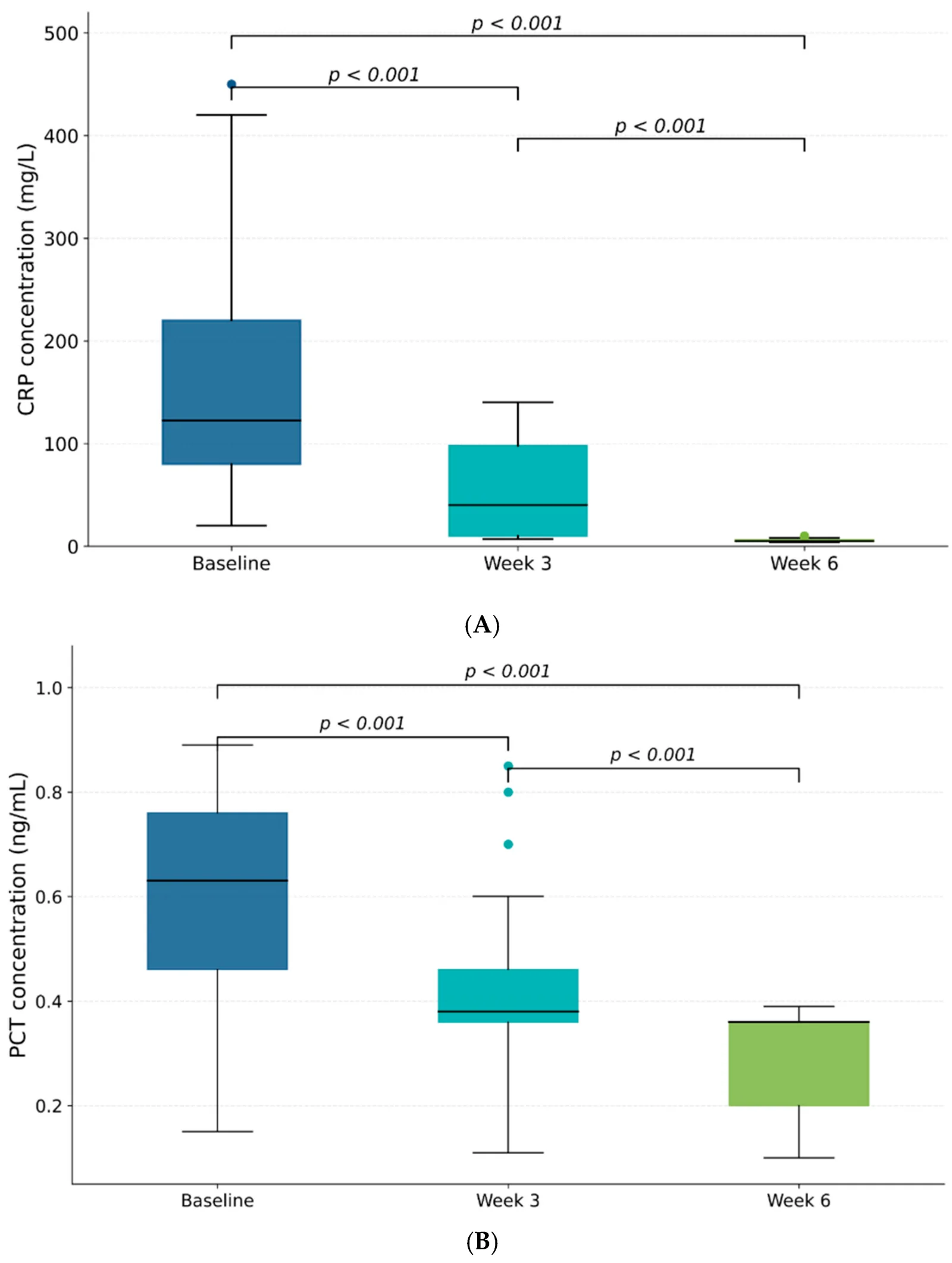
(**A**). Changes in C-reactive protein (CRP) concentration during 6-week treatment period. Statistical Analysis: Wilcoxon signed-rank test. (**B**)**.** Changes in procalcitonin (PCT) concentrations during 6-week treatment period. Statistical Analysis: Wilcoxon signed-rank test.

Significant reductions in systemic inflammatory markers were observed during follow-up. Median CRP concentrations decreased from 122.5 mg/L (IQR 80–220) at baseline to 40 mg/L (IQR 10–98) at week 3 and to 5 mg/L (IQR 5–6) at week 6. All pairwise comparisons demonstrated statistical significance (*p* < 0.001) (Figure 2A). Similarly, median PCT concentrations declined from 0.63 ng/mL (IQR 0.46–0.76) at baseline to 0.38 ng/mL (IQR 0.36–0.46) after 3 weeks and to 0.36 ng/mL (IQR 0.20–0.36) at week 6 (Figure 2B). Wilcoxon signed-rank tests confirmed statistically significant reductions in both inflammatory markers over the observation period.

At baseline, the majority of patients presented with clinical features suggestive of local infection, including oedema, erythema, purulent discharge and delayed wound healing. Progressive resolution of these findings was observed during treatment and was accompanied by improvements in wound bed appearance and exudate characteristics.

### 3.5. Microbiological Assessment

Microbiological cultures obtained at baseline confirmed a substantial infectious burden within the study cohort. Reflecting the advanced clinical presentation of CNO-associated wounds, osteomyelitis was suspected or documented in 29 of 30 patients (96.7%). The most frequently isolated pathogens included *Staphylococcus aureus*, *Pseudomonas aeruginosa*, and *Enterococcus* spp., although polymicrobial infections were also encountered.

Given the retrospective nature of the study, a uniform diagnostic pathway for osteomyelitis was not applied across all patients. Consequently, the diagnosis was established on the basis of routine clinical practice and relied on a combination of clinical findings, imaging studies, microbiological results, intraoperative observations, and specialist assessment, as available in individual cases. In addition to local signs of infection, approximately one-third of patients presented with features suggestive of advanced disease, including delayed wound healing, systemic symptoms, generalized weakness, or the presence of exposed or fragmented bone within the wound bed.

Systemic antibiotic therapy was administered whenever clinically indicated (n = 17, 56.7% of patients) and was guided by microbiological findings, infection severity, patient-specific clinical characteristics and was an integral component of the multidisciplinary treatment strategy. The most frequently prescribed agents included trimethoprim/sulfamethoxazole (n = 7, 23.3%), amoxicillin (n = 3, 10%), aminoglycosides (n = 3, 10%), clindamycin (n = 3, 10%) and cefuroxime (n = 1, 3.3%), administered either as monotherapy or in combination depending on the identified pathogens and extent of infection. Because of the retrospective real-world design, antimicrobial regimens were individualized and varied according to the identified pathogens, extent of infection, and patient-specific clinical factors. Consequently, the observed microbiological and clinical improvements should be interpreted in the context of comprehensive multidisciplinary management, including surgical debridement, antimicrobial treatment, OT and NPWTi-d.

Progressive improvement in microbiological status was observed during treatment. This was accompanied by resolution of clinical signs of infection, significant reductions in inflammatory marker concentrations, and favourable changes in wound bed appearance and exudate characteristics. By the end of the six-week observation period, follow-up microbiological findings demonstrated a lower frequency of isolation of several pathogens initially identified in wound specimens, including Pseudomonas aeruginosa, Staphylococcus aureus, and Enterococcus faecalis. These findings reflect changes in the microbiological profile of the wounds rather than quantitative reductions in bacterial load or confirmed eradication of infection. Detailed microbiological findings and treatment outcomes are presented in Table 4.

### 3.6. Exploratory Regression Analyses

Exploratory regression analyses were performed to identify baseline factors potentially associated with treatment outcomes. In models evaluating predictors of wound healing, age, body mass index, smoking status, and baseline HbA1c were not significantly associated with wound volume reduction during follow-up. Among the laboratory variables examined, only baseline CRP demonstrated a significant association with wound healing outcomes. In the exploratory multivariable model, higher baseline CRP concentrations were associated with a greater absolute reduction in estimated wound volume between baseline and week 6 (β = 0.34, 95% CI 0.17–0.50; *p* < 0.001). This association should be interpreted cautiously, particularly because absolute wound-volume reduction may be influenced by baseline wound size.

Regression models assessing factors associated with changes in pain intensity did not identify any statistically significant predictors. Baseline demographic, clinical, laboratory, and wound-related variables showed no significant associations with changes in NRS scores during follow-up.

Given the exploratory design, relatively small sample size, and absence of a control group, these findings should be interpreted with caution and regarded as hypothesis-generating rather than confirmatory.

## 4. Discussion

The present study explored short-term clinical outcomes associated with a multimodal treatment protocol incorporating OT and NPWTi-d in patients with CNO-associated foot wounds. Over the six-week observation period, we observed reductions in estimated wound volume, inflammatory biomarker levels, and pain intensity, alongside improvements in wound-bed characteristics and microbiological findings. These observations are of particular clinical interest given the complexity of the study population, including patients with substantial foot deformities, chronic ulceration, and frequently suspected bone infection. Nevertheless, these findings should be interpreted in the context of comprehensive wound management, rather than as evidence of the independent efficacy of OT or NPWTi-d.

The advanced presentation observed in our cohort likely reflects the well-recognized problem of delayed recognition and referral in CNO. Most patients were admitted after prolonged ulceration, unsuccessful previous treatment, and development of advanced complications, including osteomyelitis. Consequently, the study population represents a particularly challenging subgroup commonly encountered in specialized limb-preservation centres rather than patients with early-stage disease. Such presentations are consistent with the progressive nature of CNO, in which ongoing structural destruction may ultimately lead to deformity, recurrent ulceration, secondary infection, and increased risk of limb loss [4,22,23,24].

One of the most clinically relevant findings of the present study was the marked reduction in wound volume observed throughout follow-up. This improvement was accompanied by a transition from inflammatory and destructive wound characteristics toward granulation, tissue formation and epithelialization. Although the consistency of these observations suggests clinically meaningful wound stabilization and progression toward healing despite the advanced nature of disease at baseline, the findings should be interpreted with caution. The study cohort was relatively small, and treatment was delivered within a multimodal therapeutic framework that included surgical debridement, off-loading, systemic antimicrobial therapy, OT and NPWTi-d. Consequently, the independent contribution of individual treatment components cannot be determined, and causal inferences regarding treatment efficacy cannot be established. Nevertheless, the observed improvements may provide preliminary support for further investigation of this multidisciplinary approach in larger prospective studies.

These findings are broadly consistent with the growing body of evidence supporting the use of NPWTi-d in complex diabetic foot wounds. Recent systematic reviews and meta-analyses have demonstrated that NPWTi-d is associated with higher wound healing rates, accelerated granulation tissue formation, greater wound size reduction, and shorter time to wound closure compared with conventional wound care [17,18,25,26]. Some analyses have additionally suggested a potential reduction in amputation risk, although the quality and methodological heterogeneity of the available evidence warrant cautious interpretation [17,18]. Importantly, most published studies have focused on DFU rather than wounds associated with CNO. Consequently, evidence supporting NPWTi-d specifically in patients with CNO remains limited. One of the few studies addressing this population was reported by Ramanujam et al., who described favourable outcomes with NPWTi-d in the management of CNO-associated wounds and ankle wounds, emphasizing its role in promoting granulation tissue formation and facilitating closure of complex wounds [27]. The advanced wound characteristics observed in our cohort, including extensive tissue loss, chronic inflammation, and a high prevalence of infection, closely resemble the clinical scenarios in which NPWTi-d appears to provide the greatest benefit. Therefore, the significant reduction in wound volume and improvement in wound bed characteristics observed in the present study may further support the incorporation of NPWTi-d-based strategies within multidisciplinary limb-preservation programs for selected patients with complicated CNO.

The second major component of the treatment protocol was OT. Although the evidence base for OT remains less extensive than that for NPWTi-d, increasing attention has been directed toward its potential role in the management of chronic and infected wounds. Recent systematic reviews and meta-analyses have reported favourable effects of ozone-based interventions on wound healing, reduction in wound size, infection control, and limb preservation in patients with diabetes-related foot disease [19,20,28]. Notably, recent meta-analyses by Filho et al. and Izadi et al. demonstrated improved healing outcomes and suggested potential benefits in reducing infection burden and amputation risk, while simultaneously emphasizing the need for larger, well-designed clinical studies to strengthen the available evidence. This issue is particularly relevant in the context of our cohort, which was characterized by longstanding wounds, advanced tissue destruction, and a very high prevalence of osteomyelitis. In such patients, successful management depends not only on stimulation of tissue repair but also on effective control of infection and inflammation. The progressive improvement in microbiological findings observed during treatment may support the potential role of OT as an adjunctive component of multimodal wound management. Experimental and clinical studies suggest that ozone may exert antimicrobial effects while influencing local inflammatory responses, oxidative stress pathways, and tissue oxygen utilization within chronic wounds [19,20]. Nevertheless, because OT was administered alongside NPWTi-d, repeated debridement, off-loading, and systemic antimicrobial therapy, its independent contribution to the observed clinical improvements cannot be determined.

Particular attention should be paid to the marked reduction in CRP and PCT concentrations during follow-up. Interpretation of inflammatory biomarkers in patients with CNO requires caution. Current IWGDF guidelines do not recommend the use of CRP, white blood cell count, or other systemic inflammatory markers for diagnosing active CNO, as elevations may reflect the underlying sterile inflammatory process and demonstrate substantial variability between patients [12]. However, the clinical context of the present study differs considerably from that addressed by these recommendations. Nearly all patients in our cohort presented with chronic wounds complicated by osteomyelitis and soft tissue infection rather than isolated active CNO. In this setting, inflammatory biomarkers may provide useful adjunctive information regarding infection burden and treatment response. A recent systematic review and meta-analysis evaluating biomarkers for diabetic foot osteomyelitis reported that CRP, and PCT demonstrated acceptable to excellent diagnostic performance, with pooled area under the curve values of 0.83, 0.77, and 0.71, respectively [29]. Although none of these markers should be considered definitive diagnostic tools, their progressive decline in our cohort, together with improvement in wound appearance and microbiological findings, may reflect effective infection control during treatment. Furthermore, the observed association between higher baseline CRP concentrations and less pronounced wound volume reduction suggests that systemic inflammatory burden may influence subsequent healing outcomes in patients with advanced CNO-associated wounds.

Another clinically meaningful observation was the substantial reduction in pain intensity. Interpretation of pain outcomes in CNO is inherently challenging because advanced diabetic neuropathy may alter nociception and reduce the reliability of subjective pain assessment. Nevertheless, pain remains an important determinant of mobility, quality of life, and treatment adherence. The progressive reduction in NRS scores represents a favourable patient-reported outcome during multimodal treatment, particularly given the history of previously unsuccessful wound management. However, the heterogeneous nature of pain in CNO, together with the lack of detailed longitudinal analgesic data, precludes attribution of this improvement to infection control or any specific therapeutic intervention.

The clinical significance of these findings extends beyond wound closure alone. Ulceration and osteomyelitis are associated with an increased risk of major amputation and long-term disability in patients with diabetic foot disease, highlighting the importance of limb-preservation strategies, particularly in complex cases involving CNO [3,30]. Consequently, contemporary treatment strategies increasingly focus on limb preservation rather than wound healing as an isolated endpoint. The favourable outcomes observed in the present study should therefore be interpreted within the broader context of preserving function, maintaining ambulation, and preventing progression to major amputation.

Current IWGDF recommendations emphasize that successful management of complex DFD complications requires integration of wound care specialists, podiatrists, orthopaedic surgeons, infectious disease physicians, vascular specialists, and rehabilitation teams [12]. Such a multidisciplinary approach has become increasingly important in patients with CNO because isolated wound management rarely addresses the underlying biomechanical, infectious, and structural factors responsible for disease progression. The treatment protocol evaluated in the present study was implemented within a multidisciplinary limb-preservation framework, consistent with contemporary approaches to the management of complicated diabetic foot wounds.

The present study possesses several strengths. To our knowledge, reports evaluating the combined use of OT and NPWTi-d specifically in patients with CNO-associated wounds remain scarce. Furthermore, the analysed cohort represents a particularly challenging population characterized by advanced deformity, chronic ulceration, osteomyelitis, and multiple comorbidities. Consequently, the study provides valuable real-world evidence derived from routine specialist wound care practice.

An important consideration when interpreting the present findings is that local wound improvement does not necessarily indicate correction of the underlying biomechanical abnormalities associated with CNO. Although surgical offloading procedures, including minimally invasive metatarsal osteotomies, have demonstrated favourable outcomes in selected patients with neuropathic forefoot ulcers, their applicability to CNO-associated wounds depends on the anatomical pattern and severity of deformity. In patients with substantial midfoot collapse or mechanical instability, more extensive reconstructive procedures may be required. Therefore, the short-term reductions in wound volume observed in our cohort should not be interpreted as evidence of durable biomechanical correction or reduced long-term ulcer recurrence. These outcomes require longer follow-up and should be evaluated separately from the effects of local wound management.

Several limitations should also be acknowledged. The retrospective design, single-centre setting, relatively small sample size, and absence of a control group limit causal interpretation of the findings. Because treatment consisted of multiple simultaneously applied interventions, including surgical debridement, systemic antimicrobial therapy, off-loading, OT, and NPWTi-d, the independent effects of individual treatment components cannot be established. Furthermore, detailed CNO staging, disease activity, anatomical classification, deformity characteristics, disease duration, previous Charcot-related surgery, and adherence to offloading were not consistently documented, limiting assessment of clinical heterogeneity and its potential influence on treatment outcomes. Consequently, the observed improvements cannot be attributed specifically to the combination of OT and NPWTi-d, and the present study does not establish an additive or synergistic treatment effect. In addition, diagnostic pathways for osteomyelitis were not fully standardized because data were derived from routine clinical practice. Given the individualized diagnostic approach to osteomyelitis, differences in the degree of diagnostic certainty should be considered when interpreting infection-related outcomes. Furthermore, the six-week observation period was primarily intended to assess short-term wound-related changes rather than long-term resolution of bone infection. It was sufficient to characterize early wound-related changes but did not permit assessment of sustained wound healing, ulcer recurrence, subsequent amputations, or long-term limb preservation. Therefore, the observed improvements should be interpreted as short-term clinical outcomes rather than evidence of durable treatment effectiveness as well as considered exploratory and hypothesis-generating rather than confirmatory.

What is more, the relatively small sample size also limits the robustness of exploratory regression analyses, potentially affecting the stability of coefficient estimates; consequently, the observed associations should be considered hypothesis-generating and require validation in larger cohorts.

Future prospective controlled studies are required to determine whether ozone-based NPWTi-d protocols provide clinically meaningful advantages over contemporary standard wound care approaches in patients with CNO complicated by chronic ulceration and infection.

## 5. Conclusions

In this retrospective case series, a multidisciplinary treatment strategy incorporating OT and NPWTi-d was associated with favourable short-term changes in estimated wound volume, inflammatory markers, pain intensity, and wound microbiological findings in patients with CNO-associated foot wounds. Given the absence of a control group, concurrent therapeutic interventions, and limited follow-up, these observations do not establish treatment-specific efficacy, confirmed infection eradication, or long-term limb preservation. Nevertheless, they provide a rationale for further evaluation of this multimodal approach in prospective controlled studies.

## Figures and Tables

**Table 1:** General characteristics of the study cohort.

	Mean (±SD)/n (%)
**Age (years)**	64.2 (±16.6)
**Sex (M/F)**	22 (73.3)/8 (26.7)
BMI * (kg/m^2^)	27.8 (±5.2)
DM * type 1/DM type 2	20 (66.7)/10 (33.3)
**CARDIOVASCULAR:**	
- chronic coronary syndrome	14 (46.7)
- myocardial infarction in history	11 (36.7)
- chronic heart insufficiency	8 (26.7)
- arrhythmias	17 (56.7)
- arterial hypertension	24 (80)
- chronic venous insufficiency	7 (23.3)
**HAEMATOLOGIC:**	
- anaemia	24 (80)
- leukopenia	3 (10)
- leukaemia	0 (0)
- lymphoma	0 (0)
**PULMONARY:**	
- asthma	9 (30)
**HYPOTHYROIDISM**	0 (0)
**HYPERTHYROIDISM**	6 (20)
**GASTROINTESTINAL:**	
- gastric ulcer disease	7 (23.3)
- duodenal ulcer disease	7 (23.3)
- cholelithiasis	7 (23.3)
- gastroesophageal reflux disease	19 (63.3)
- chronic diarrhoea	1 (3.3)
**HEPATIC CIRRHOSIS**	5 (16.7)
**NEPHROTIC PROTEINURIA**	1 (3.3)
**URINARY INCONTINENCE**	13 (43.3)
**URINARY TRACT NEOPLASMS**	4 (13.3)
**GOUT**	7 (23.3)
**NEUROLOGICAL:**	
- peripheral neuropathy	26 (86.7)
- polyneuropathy	26 (86.7)
- paraplegia	1 (3.3)
- tetraplegia	0
- depression	16 (53.3)

* BMI—body mass index; DM—diabetes mellitus.

**Table 2:** Clinical evaluation of ulcerations before initiation of therapy.

Clinical Evaluation (A)	n (%)
**Duration of ulcer:**	
- One year or more	20 (66.7)
- One month or more	10 (33.3)
**Duration of treatment to date:**	
- One year or more	12 (40)
- One month or more	14 (46.7)
- One week or more	4 (13.3)
**Location of ulcer:**	
- On the plantar surface	30 (100)
- On the forefoot	0 (0)
- On the hallux (big toe)	0 (0)
- On toes II, III, IV, V	0 (0)
**Other characteristic features:**	
- No pain or mild pain	4 (13.3)
- Sensory disturbances	28 (93.3)
- Foot normally warm and pink	3 (10)
- Palpable pulse in the foot	23 (76.7)

**Table 3:** Therapeutic approaches for CNO-associated wounds prior to study enrolment.

Method	n (%)
**DISINFECTION:**	30 (100)
- Octenidine	27 (90)
- Polyhexanide	6 (20)
- Povidone–iodine solution	10 (33.3)
- Sodium hypochlorite	0 (0)
**WOUND DEBRIDEMENT** (scalpel or surgical spoon)	5 (16.7)
**OINTMENTS:**	30 (100)
- Betadine	16 (53.3)
- Iruxol	14 (46.7)
**GELS FOR WOUND DEBRIDEMENT**:	17 (56.7)
- Granudacyn	14 (46.7)
- Microdacyn	2 (6.7)
- Intrasite	1 (3.3)
**DRESSINGS:**	30 (100)
HYDROCOLLOID DRESSINGS:	10 (33.3)
- Granuflex	8 (26.7)
- Hydrocoll	2 (6.7)
DRESSINGS SILVER ION	30 (100)
MANUKA HONEY DRESSINGS	0 (0)
IODINE-CONTAINING INADINE DRESSING	4 (13.3)
ABSORBENT DRESSINGS	30 (100)
- Gauze and sterile compresses	24 (80)
- Zetuvit	17 (56.7)
- Mepilex	11 (36.7)
- Aquacel Foam	9 (30)
- Mepilex Border	5 (16.7)
- Suprasorb	2 (6.7)
- Sorbact	2 (6.7)
- Biatain	1 (3.3)
**OTHER:**	0 (0)
- Biological dressings (larval therapy)	0 (0)
- Negative pressure wound therapy	0 (0)
- Ozone therapy	0 (0)
- Hyperbaric chamber treatment	0 (0)

**Table 4:** Descriptive comparison of microbiological findings in CNO-associated foot wounds at baseline and week 6.

Microbiological Finding	Baseline (0) n (%)	Week 6 n (%)
Staphylococcus aureus	21 (70)	1 (3.3)
Pseudomonas aeruginosa	26 (86.7)	2 (6.7)
Staphylococcus lugdunensis	2 (6.7)	0 (0)
Streptococcus agalactiae	4 (13.3)	0 (0)
Streptococcus dysgalactiae	0 (0)	0 (0)
Proteus mirabilis	6 (20)	0 (0)
Enterococcus faecalis	25 (83.3)	0 (0)
Escherichia coli	4 (13.3)	1 (3.3)
Granulicatella adiacens	1 (3.3)	0 (0)
Enterobacter cloacae	2 (6.7)	0 (0)
Staphylococcus aureus MRSA	3 (10)	0 (0)
Citrobacter koseri	1 (3.3)	0 (0)

Data are presented as n (%) of patients with each microorganism identified in wound cultures at baseline and week 6 (n = 30 at each time point). Multiple microorganisms could be isolated from an individual patient.

## Data Availability

The datasets generated and/or analyzed during the current study are not publicly available due to patient confidentiality, data protection requirements, and institutional restrictions governing access to clinical records. The data are held by the institution responsible for their collection and cannot be independently shared by the authors.

## Abbreviations

The following abbreviations are used in this manuscript:

BMI	body mass index
CRP	C-reactive protein
DFD	diabetes-related foot disease
DFU	diabetic foot ulcer
CNO	Charcot neuro-osteoarthropathy
DM	diabetes mellitus
HBA1c	glycated haemoglobin
NPWTi-d	negative pressure wound therapy with instillation and dwell time
NRS	numeric rating scale
OT	ozone therapy
PCT	procalcitonin
SD	standard deviation
TcPO_2_	transcutaneous oxygen pressure
